# Genotype-environment interaction and stability of fiber properties and growth traits in triploid hybrid clones of *Populus tomentosa*

**DOI:** 10.1186/s12870-021-03156-6

**Published:** 2021-09-06

**Authors:** Jian Wu, Qing Zhou, Yaru Sang, Xiangyang Kang, Pingdong Zhang

**Affiliations:** 1 Beijing Advanced Innovation Center for Tree Breeding by Molecular Design, Beijing Forestry University, Beijing, 100083 China; 2 National Engineering Laboratory for Tree Breeding, Beijing Forestry University, Beijing, 100083 China; 3 Key Laboratory of Genetics and Breeding in Forest Trees and Ornamental Plants, Ministry of Education, Beijing Forestry University, Beijing, 100083 China; 4 College of Biological Sciences and Technology, Beijing Forestry University, Beijing, 100083 China

**Keywords:** Genotype-environment interactions, Stability parameter, Clonal repeatability, fiber property, Growth trait, *P. tomentosa*

## Abstract

**Background:**

Clones provide a sensitive method for evaluating genotypic stability and detecting genotype-environment (G × E) interactions because of non-additive genetic effects among clones and there being no genetic effect among ramets of an ortet. With this study, we aimed to confirm and expand earlier findings, estimate stability parameters, and provide accurate estimates of clonal repeatabilities and genetic gains for a triploid breeding program of *P. tomentosa* Carr.

**Results:**

Six 5-year-old clonal trials established in Northern China were used to determine the clonal variation, clone × site interactions, and the stability parameters of fiber properties of wood and growth traits. 360 trees from ten hybrid clones were collected from six sites. The clonal and site effects had a highly significant effect (*P* < 0.001) for all studied traits. While the clone × site interactions had a highly significant effect (*P* < 0.001) on fiber length (FL), coarseness (C), and tree growth (tree height [H], diameter at breast height [DBH] and stem volume [SV]), and a moderate effect (*P* < 0.05) on fiber width (FW) and fiber length/width (FL/W). For FL and SV, most of the triploid hybrid clones had higher reaction norms to the improvement in growth conditions and higher phenotypic plasticity. The estimated clonal repeatability of FW (0.93) was slightly higher than for FL (0.89), FL/W (0.83), C (0.91), DBH (0.76), H (0.85), and SV (0.80). Three clonal testing sites were sufficient to estimate quantitative parameters of fiber properties, however, more clonal testing sites would help improve the accuracy of quantitative parameters of the growth traits.

**Conclusions:**

Our results highlight that accurate estimation of quantitative parameters for growth traits in triploid hybrid clones of *P. tomentosa* requires more clonal testing sites than the fiber properties.

## Background

Chinese white poplar, *P. tomentosa* Carr. (section Populus, family Salicaceae, genus *Populus*), is a widely distributed tree species in northern China and generally grows in mixed-species forests with *Robinia pseudoacacia* L. Since the first triploid hybrid of *P. tomentosa* was artificially created in 1995, the triploid hybrid clones have exhibited superior growth in China [1]. The latest research progress in wood fiber has led to the use of short fibers and short fibers mixed with coniferous long fibers for the production of high-quality paper [2]. The Chinese forestry industry has shown a new focus on using hybrid poplar for manufacturing evenly short fibers. Thus, an obvious increase in Chinese white poplar-use is expected, which in turn increases interest in breeding selected triploid hybrid clones [3–5].

The triploid hybrid clones of *P. tomentosa* have been shown to display a high degree of variability in growth, fiber traits, wood basic density, and wood chemical properties [1, 3–6]. This variability indicates that intriguing gains may be obtained through selection. The potential advantages of utilizing clonal methods in poplar hybrid clones have been reviewed by several authors [7, 8]. Genotype × environment (G × E) interaction normally refers to the performance differences of genotypes between environments [9]. Testing and selection in tree improvement programs are complicated by this interaction, and it often leads to reduced total genetic gains. In general, the literature on G × E is plentiful for forest trees [10, 11], however, the number of studied clones is relatively limited [12]. It is expected that clones are more sensitive in different environments than either seed- or family-origin plants because there is no genetic effects among ramets of an ortet and non-additive genetic effects among clones are substantial. Therefore, clones can provide a sensitive method for evaluating genotypic stability and detecting G × E interactions [13].

To obtain a better understanding of the genetic control of growth traits, fiber properties, wood basic density and wood chemical properties, basic genetics such as G × E interactions, inter-trait and inter-site correlations, clonal genetic variation, and repeatabilities for triploid hybrid clones of *P. tomentosa* were recently estimated [4, 5, 14, 15]. Both site effects and clonal effects were found to be significant for all tested traits and G × E interactions were also significant for all studied traits except for fiber length and holocellulose [5, 14]. This basic information is crucial to estimate expected genetic gains and develop suitable strategies for clonal forestry. However, most of the studies were conducted on only three or four sites. For the purpose of tree improvement, accurate estimates of quantitative parameters require that different genotypes are tested at more locations, therefore, in this present study we study six sites.

The objectives of the present study were: (1) to confirm and expand the earlier findings; (2) to estimate stability parameters of these triploid clones; and (3) to provide accurate estimated values of genetic gains and clonal repeatabilities for improving the breeding program of Chinese white poplar.

## Results

### Basic statistics and variation within and between sites

The mean values, standard error (SE) of the mean, ranges of maximum and minimum values, and coefficients of phenotypic variation of all traits in the six clonal trials are presented in Table 1. A fairly obvious difference in growth was found between the six locations, with trees at the Taiyuan site exhibiting the fastest growth. For fiber properties, the longest fiber length (FL) and the greatest fiber length/width ratio (FL/W) were found in the trees at the Gaotang site. Trees from the Pinggu site had the shortest FL and the lowest FL/W. The difference between the longest and shortest means of FL was 7.9% (Table 1). However, the thinnest fibers were observed at Xiangfen, and the thickest, at Pinggu. The combined analysis of all six trials displayed that all studied traits exhibited significant site effects (Table 2). FL, fiber width (FW), and FL/W showed small phenotypic variation (CV = 3.2–5.5%) and the values were much lower than those of coarseness (C), diameter at breast height (DBH; at 1.3 m above ground level), tree height (H), and stem volume (SV).

**Table 1:** Clone mean values, standard error (SE) of the mean, ranges of maximum and minimum values, and coefficients of phenotypic variation (CV *p* %) for fiber properties and growth traits at the six clonal trials

Site	Traits	Triploid clones			Diploid clone (M1319)	
		Mean ± SE	Range (min-max)	CV p %	Mean ± SE	Range (min-max)
Yanzhou	FL (mm)	0.77 ± 0.04	0.68 ± 0.84	5.3	0.67 ± 0.01	0.65 ± 0.69
	FW (μm)	24.7 ± 0.9	23.2 ± 26.6	3.5	22.7 ± 1.2	21.9 ± 25.0
	FL/W	31.3 ± 1.4	28.4 ± 33.4	4.5	29.4 ± 1.1	27.6 ± 30.7
	C (μg/m)	105.8 ± 8.9	86.5 ± 124.3	8.4	91.8 ± 10.9	83.3 ± 113.4
	H (m)	12.5 ± 1.2	10.6 ± 15.4	9.7	9.9 ± 0.5	9.2 ± 10.6
	DBH (cm)	10.7 ± 1.0	8.5 ± 13.3	9.4	9.0 ± 1.2	7.1 ± 10.4
	SV (dm3)	48.2 ± 11.0	26.2 ± 70.4	22.9	28.7 ± 8.2	16.4 ± 39.5
Gaotang	FL (mm)	0.82 ± 0.04	0.73 ± 0.90	4.9	0.69 ± 0.03	0.64 ± 0.74
	FW (μm)	24.2 ± 0.9	22.6 ± 26.0	3.6	22.1 ± 0.5	21.5 ± 22.7
	FL/W	33.9 ± 1.5	30.7 ± 37.1	4.3	31.1 ± 1.2	29.7 ± 32.6
	C (μg/m)	102.2 ± 7.4	85.7 ± 118.9	7.3	85.5 ± 3.0	82.5 ± 89.0
	H (m)	13.6 ± 1.2	9.4 ± 16.0	8.9	12.0 ± 0.6	11.1 ± 12.9
	DBH (cm)	11.6 ± 1.5	7.8 ± 14.9	12.9	9.7 ± 0.8	8.6 ± 10.8
	SV (dm3)	61.8 ± 18.4	23.6 ± 113.9	29.8	38.6 ± 7.3	28.1 ± 48.9
Xiangfen	FL (mm)	0.80 ± 0.04	0.73 ± 0.90	5.0	0.67 ± 0.02	0.64 ± 0.71
	FW (μm)	24.2 ± 0.9	22.6 ± 26.0	3.7	22.0 ± 0.7	21.3 ± 22.9
	FL/W	33.1 ± 1.1	30.3 ± 35.4	3.4	30.4 ± 1.6	28.1 ± 32.7
	C (μg/m)	99.6 ± 9.3	68.6 ± 120.3	9.3	82.0 ± 5.7	76.5 ± 92.4
	H (m)	13.2 ± 1.1	11.4 ± 15.8	8.4	11.5 ± 0.4	10.8 ± 12.1
	DBH (cm)	11.7 ± 1.2	9.0 ± 14.6	10.3	10.0 ± 0.7	8.8 ± 10.7
	SV (dm3)	60.8 ± 15.6	31.5 ± 107.1	25.7	39.4 ± 5.7	28.8 ± 46.4
Pinggu	FL (mm)	0.76 ± 0.04	0.67 ± 0.85	5.3	0.61 ± 0.02	0.58 ± 0.63
	FW (μm)	26.1 ± 1.0	23.7 ± 28.8	3.8	22.9 ± 0.2	22.5 ± 23.1
	FL/W	29.1 ± 1.6	25.8 ± 31.7	5.5	26.5 ± 0.9	25.2 ± 28.1
	C (μg/m)	112.5 ± 7.8	97.8 ± 129.4	6.9	85.2 ± 2.2	80.9 ± 87.2
	H (m)	11.4 ± 1.5	7.8 ± 15.8	13.2	10.6 ± 0.9	9.1 ± 11.8
	DBH (cm)	13.8 ± 2.2	8.7 ± 19.3	15.9	10.4 ± 1.3	8.2 ± 11.9
	SV (dm3)	75.3 ± 28.0	21.1 ± 133.4	37.2	39.8 ± 10.6	21.3 ± 52.9
Zhengzhou	FL (mm)	0.81 ± 0.04	0.74 ± 0.89	4.9	0.68 ± 0.03	0.64 ± 0.72
	FW (μm)	25.3 ± 0.8	23.5 ± 27.1	3.2	22.2 ± 0.6	21.6 ± 23.1
	FL/W	32.2 ± 1.5	28.8 ± 34.8	4.7	30.6 ± 1.1	29.1 ± 32.2
	C (μg/m)	111.2 ± 8.6	88.2 ± 127.5	7.7	87.5 ± 3.8	82.3 ± 91.4
	H (m)	13.0 ± 1.5	9.2 ± 15.1	11.5	11.1 ± 0.8	10.4 ± 12.6
	DBH (cm)	14.4 ± 1.8	9.8 ± 18.5	12.5	12.3 ± 1.0	11.1 ± 13.6
	SV (dm3)	91.0 ± 26.7	32.9 ± 152.7	29.3	57.2 ± 12.2	44.3 ± 75.5
Taiyuan	FL (mm)	0.78 ± 0.04	0.72 ± 0.87	5.1	0.65 ± 0.03	0.61 ± 0.68
	FW (μm)	24.6 ± 0.9	22.6 ± 27.4	3.7	21.6 ± 0.4	21.0 ± 22.1
	FL/W	31.7 ± 1.1	29.0 ± 34.2	3.5	29.9 ± 1.4	27.9 ± 31.4
	C (μg/m)	103.5 ± 8.4	84.4 ± 120.0	8.1	80.0 ± 1.6	77.7 ± 81.8
	H (m)	13.9 ± 1.7	9.5 ± 16.4	12.2	11.7 ± 0.5	11.0 ± 12.2
	DBH (cm)	14.8 ± 1.9	11.3 ± 18.5	12.8	13.0 ± 0.8	11.7 ± 13.6
	SV (dm3)	100.9 ± 31.1	46.1 ± 172.4	30.8	65.5 ± 9.2	50.7 ± 74.6

**Table 2:** Analysis of variance results, estimates of repeatability (± SE) at the clonal mean and individual-tree levels of all traits of the nine triploid hybrid clones at the six sites combined

Traits	*P*-value			${R}_b^2$	${R}_c^2$	Percentage (%)
	Clones	Sites	Sites × clones			
FL	0.000	0.000	0.000	0.29 ± 0.11	0.89 ± 0.05	73.9
FW	0.000	0.000	0.031	0.36 ± 0.13	0.93 ± 0.03	87.6
FL/W	0.000	0.000	0.014	0.17 ± 0.08	0.83 ± 0.07	68.4
C	0.000	0.000	0.000	0.35 ± 0.13	0.91 ± 0.04	72.7
H	0.000	0.000	0.001	0.18 ± 0.09	0.76 ± 0.09	42.1
DBH	0.000	0.000	0.001	0.21 ± 0.10	0.85 ± 0.06	66.2
SV	0.000	0.000	0.000	0.19 ± 0.09	0.80 ± 0.08	52.9

Note: Estimated percentage ratio (%) of clonal variance component to the total variance component of clone×site interaction and clone. The degrees of freedom are 5 for the site, 8 for the clone, 40 for the clone × interaction, and 270 for the error

### Clonal variation and repeatability

The results of the analysis of variance for the combined six sites are presented in Table 2. Significant clonal effects for all tested traits were seen (Table 2). For all studied traits, the variance caused by error (that is, the differences between ramets within a clone within a site) accounted for most of the variation (48.4 to 75.4% of the overall variation). However, most of the variance in FW (35.7%) was caused by the clone. Hence, the highest estimated repeatability of the clone mean (0.93) and the estimated individual-tree clonal repeatability (0.36) were observed for the FW.

The estimated clonal repeatability and estimated individual-tree clonal repeatability of all the studied traits at each site are shown in Table 3. No significant differences were found between the clones for H, DBH, and SV at the Yanzhou site, FL/W at the Gaotong site, as well as FW and FL/W at the Pinggu site. Therefore, we did not estimate the clonal repeatability for the six traits. The estimated clonal repeatability varied from 0.73 to 0.90 for FL, from 0.68 to 0.95 for FW, from 0.54 to 0.90 for FL/W, and from 0.78 to 0.93 for C. The estimated clonal repeatability of the growth traits ranged from 0.58 to 0.90 (Table 3).

**Table 3:** The results of analysis of variance and estimates of repeatability of all studied traits of the nine triploid hybrid clones at individual site

Site		Trait						
		FL	FW	FL/W	C	H	DBH	SV
Yanzhou	*P* value	0.000	0.000	0.000	0.000	0.389	0.516	0.786
	${R}_b^2$	0.58	0.57	0.60	0.53	–	–	–
	${R}_c^2$	0.90 ± 0.05	0.95 ± 0.03	0.90 ± 0.07	0.87 ± 0.08	–	–	–
Gaotang	P value	0.000	0.000	0.054	0.000	0.000	0.000	0.000
	${R}_b^2$	0.49	0.46	–	0.70	0.45	0.44	0.43
	${R}_c^2$	0.85 ± 0.07	0.84 ± 0.10	–	0.93 ± 0.05	0.83 ± 0.08	0.82 ± 0.08	0.82 ± 0.08
Xiangfen	P value	0.000	0.000	0.041	0.000	0.000	0.020	0.005
	${R}_b^2$	0.38	0.54	0.17	0.44	0.39	0.21	0.29
	${R}_c^2$	0.79 ± 0.09	0.88 ± 0.08	0.56 ± 0.20	0.83 ± 0.11	0.79 ± 0.09	0.61 ± 0.14	0.70 ± 0.12
Pinggu	P value	0.000	0.073	0.062	0.000	0.030	0.000	0.001
	${R}_b^2$	0.45	–	–	0.45	0.19	0.38	0.33
	${R}_c^2$	0.83 ± 0.08	–	–	0.83 ± 0.08	0.58 ± 0.15	0.78 ± 0.10	0.74 ± 0.11
Zhengzhou	P value	0.002	0.000	0.020	0.000	0.000	0.030	0.014
	${R}_b^2$	0.31	0.38	0.21	0.37	0.47	0.19	0.23
	${R}_c^2$	0.73 ± 0.11	0.79 ± 0.10	0.61 ± 0.14	0.78 ± 0.10	0.84 ± 0.07	0.58 ± 0.15	0.64 ± 0.14
Taiyuan	P value	0.000	0.007	0.047	0.000	0.000	0.000	0.000
	${R}_b^2$	0.42	0.26	0.17	0.64	0.61	0.39	0.48
	${R}_c^2$	0.81 ± 0.09	0.68 ± 0.13	0.54 ± 0.16	0.91 ± 0.04	0.90 ± 0.05	0.79 ± 0.09	0.85 ± 0.07

Note: The degree of freedom for the clone is 8 and 45 for the error

### Clone × site interaction, stability, and reaction norms of triploid hybrid clones

In this study, all fiber properties and growth traits exhibited a significant clone × site interaction (Table 2). The level of significance for FW (0.031) and FL/W (0.014) was moderate and a highly significant effect (*P* < 0.001) was observed for FL, C, and tree growth. A higher estimated ratio in H (57.9%) for the clone × site interaction variance component versus the total clone×site plus clone variance component was observed compared with the other traits.

The inter-site (B-type) genotypic correlations are shown in Table 4. Some inter-site genotypic correlations between the same traits were not estimated because no significant differences were observed between the clones for H, DBH, and SV at the Yanzhou site, FL/W at the Gaotong site, and FW and FL/W at the Pinggu site. Most of the inter-site genotypic correlations between the same traits at different sites were either moderate or strong, except for H at Zhengzhou-Taiyuan and FL at Pinggu-Taiyuan (Table 4). For FL, FL/W, and the growth traits, the correlations were lower when the Taiyuan site was included.

**Table 4:** The estimates of type B genetic correlations among all possible pairs of sites for fiber properties and growth traits (with approximate SE in brackets) of the nine triploid hybrid clones

Pairs of site	Genetic correlation						
	FL	FW	FL/W	C	H	DBH	SV
YZ-GT	0.91(0.01)*	0.97(0.01)*	–	0.63(0.03)	–	–	–
YZ-XF	0.90(0.01)*	0.59(0.02)	0.67(0.03)	0.58(0.05)	–	–	–
YZ-PG	0.92(0.01)*	–	–	0.91(0.01)*	–	–	–
YZ-ZZ	0.68(0.04)	0.86(0.01)*	1.13(0.01)**	0.70(0.02)	–	–	–
YZ-TY	0.12(0.05)	1.01(0.01)**	0.20(0.09)	0.54(0.02)	–	–	–
GT-XF	0.98(0.01)*	0.90(0.01)*	–	0.55(0.04)	0.69(0.03)	0.57(0.07)	0.51(0.07)
GT-PG	0.99(0.01)*	–	–	0.74(0.01)	1.32(0.04)**	0.96(0.01)*	0.98(0.01)*
GT-ZZ	0.91(0.01)*	0.96(0.01)*	–	0.67(0.02)	0.88(0.01)*	1.13(0.02)**	1.01(0.01)**
GT-TY	0.35(0.06)	1.03(0.01)**	–	0.66(0.01)	0.16(0.04)	0.61(0.03)	0.44(0.04)
XF-PG	0.90(0.01)*	–	–	1.05(0.01)**	1.06(0.01)**	0.91(0.01)*	0.84(0.02)
XF-ZZ	1.05(0.01)**	0.79(0.02)	0.84(0.03)	1.16(0.01)**	0.79(0.02)	1.25(0.04)**	1.08(0.01)**
XF-TY	0.26(0.07)	0.56(0.04)	0.01(0.01)	0.70(0.02)	0.45(0.03)	0.22(0.09)	0.19(0.07)
PG-ZZ	0.96(0.01)*	–	–	1.10(0.01)**	1.13(0.01)**	1.27(0.03)**	1.26(0.03)**
PG-TY	0.08(0.07)	–	–	0.68(0.02)	0.55(0.04)	0.66(0.03)	0.60(0.03)
ZZ-TY	0.28(0.08)	0.96(0.01)*	0.64(0.07)	0.64(0.02)	−0.02(0.01)	0.59(0.05)	0.36(0.06)

Note: *Significant correlations *p* < 0.05. **denotes *p* < 0.01

The stability parameters for FL and SV of the individual triploid hybrid clones across the six clonal trials, including the Finlay-Wilkinson parameters and the Shukla stability variances that characterize the reaction norms of the clones are shown in Table 5. The stability parameters of all of the studied clones were estimated only for FL and SV. For FL, the Shukla’s stability of clones B330 and B331 was slightly higher than those of the other clones due to their lower stability variance (1.01 × 10 ^− 4^ and 2.28 × 10 ^− 4^, respectively). However, for SV, lower stability variances were observed in clones B302, B303, and B306. Therefore, clones B302, B303, and B306 had higher stability for SV than the other clones. For FL, using the Finlay-Wilkinson’s method, the regression coefficient (*b*_*i*_) of clones B302, B303, B304, and B306 varied from 1.214 to 1.429, indicating that the four clones had higher reaction norms. However, the clones, B304, B330, and B331 had higher reaction norms for SV because their regression coefficients (*b*_*i*_) were 1.315, 1.411, and 1.477, respectively.

**Table 5:** Stability parameters for FL and SV of the nine triploid hybrid clones across six sites

Traits	Clones	Shukla stability variance			Finlay-Wilkinson stability parameters			
		Variance × 10^−4^	*F*	*P*	Intercept	*t*	*bi*	*t*
FL	B301	4.31	3.14	0.009	0.068	0.22	0.929	2.41
	B302	4.56	3.33	0.006	−0.241	− 0.91	1.321	3.97
	B303	3.80	2.77	0.018	−0.188	− 0.68	1.214	3.49
	B304	4.06	2.96	0.013	− 0.204	− 0.74	1.250	3.59
	B305	6.34	4.62	0.000	0.043	0.12	0.893	1.91
	B306	6.59	4.81	0.000	−0.315	−0.94	1.429	3.37
	B312	5.83	4.25	0.001	0.193	0.58	0.750	1.79
	B330	1.01	0.74	0.594	0.115	0.80	0.893	4.93
	B331	2.28	1.66	0.143	0.246	1.35	0.679	2.95
SV	B301	3.16	5.39	0.000	18.673	0.78	0.667	2.10
	B302	0.56	0.95	0.448	11.26	0.73	0.870	4.27
	B303	0.95	1.63	0.153	13.233	0.83	0.908	4.28
	B304	2.43	4.14	0.001	−25.668	−1.11	1.315	4.26
	B305	2.02	3.44	0.005	14.072	1.12	0.466	2.79
	B306	0.72	1.22	0.298	0.923	0.07	0.941	5.07
	B312	1.23	2.11	0.065	13.508	0.74	0.919	3.78
	B330	2.12	3.61	0.004	−26.084	−1.39	1.411	5.64
	B331	2.04	3.48	0.005	−19.436	− 1.22	1.477	7.00

Note: *F*: Fisher’s criteria; *P*: probability levels; *t*: Student’s criteria; *b*_*i*_: regression coefficient

### Inter-trait genetic correlations, correlated genetic response, and genetic gain

The genotypic and phenotypic correlations between all of the studied traits at individual sites are presented in Table 6. At all sites, a positive estimated correlation was found between FL and growth traits. A significant positive estimated genetic correlation between FW and C was also observed at each site. However, a negative estimated genotypic correlation between growth traits (except for H) and the C existed. These results suggest that selection for growth traits (except for H) might lead to a slight decrease in C. SV, H, and DBH had some strong positive autocorrelations since SV was derived from H and DBH.

**Table 6:** Phenotypic correlations (below the diagonal) and genotypic correlations (above the diagonal) among all traits at individual sites of the nine triploid hybrid clones

Site	Traits	FL	FW	FL/W	C	H	DBH	SV
Yanzhou	FL		0.59	0.77*	0.87*	–	–	–
	FW	0.55		−0.25	0.97*	–	–	–
	FL/W	0.69*	− 0.23		0.16	–	–	–
	C	0.77*	0.88*	0.14		–	–	–
	H	0.10	−0.14	0.24	− 0.29		–	–
	DBH	0.26	− 0.34	0.59	− 0.05	0.24		–
	SV	0.29	−0.49	0.75*	−0.12	0.74*	0.90**	
Gaotang	FL		0.92*	–	0.71	0.65	0.32	0.35
	FW	0.78*		–	0.99**	0.25	−0.19	−0.16
	FL/W	0.68*	0.08		–	–	–	–
	C	0.63	0.88**	0.04		−0.01	−0.34	−0.30
	H	0.55	0.21	0.64	−0.01		0.82*	0.93*
	DBH	0.27	−0.16	0.60	−0.30	0.68*		1.20**
	SV	0.29	−0.13	0.61	−0.26	0.77*	0.98**	
Xiangfen	FL		0.96**	0.63	0.84*	0.52	0.66	0.56
	FW	0.80**		−0.27	0.96**	−0.01	0.07	0.03
	FL/W	0.42	−0.19		−0.19	1.04*	1.16*	1.03*
	C	0.68*	0.82**	−0.13		− 0.32	−0.11	− 0.21
	H	0.41	−0.01	0.69*	−0.26		1.37**	1.31**
	DBH	0.46	0.05	0.68*	−0.08	0.95**		1.51**
	SV	0.42	0.02	0.65	−0.16	0.98**	0.99**	
Pinggu	FL		–	–	0.75*	1.10**	0.56	0.62
	FW	0.49		–	–	–	–	–
	FL/W	0.81**	−0.11		–	–	–	–
	C	0.68*	0.86**	0.20		0.40	−0.19	−0.16
	H	0.88**	0.17	0.90**	0.31		1.05**	1.11**
	DBH	0.51	−0.25	0.80*	−0.18	0.80**		1.16**
	SV	0.54	−0.24	0.83**	−0.15	0.83**	0.99**	
Zhengzhou	FL		0.63	0.89*	0.68	0.49	0.46	0.43
	FW	0.54		−0.33	0.93**	−0.23	−0.52	− 0.53
	FL/W	0.68*	−0.26		−0.06	0.85*	1.10*	1.07*
	C	0.58	0.82**	−0.05		0.29	−0.20	− 0.10
	H	0.44	−0.21	0.69*	0.26		0.97*	1.08**
	DBH	0.34	−0.40	0.74*	−0.15	0.76*		1.42**
	SV	0.34	−0.42	0.75*	−0.08	0.88**	0.97**	
Taiyuan	FL		0.97**	1.01*	0.83**	0.33	0.24	0.34
	FW	0.82**		0.36	1.01**	0.17	−0.28	−0.11
	FL/W	0.76*	0.25		0.43	0.47	0.84	0.83
	C	0.82**	0.91**	0.34		−0.05	−0.23	− 0.15
	H	0.32	0.15	0.37	−0.05		0.67	0.82*
	DBH	0.21	−0.23	0.61	−0.22	0.62		1.09**
	SV	0.31	−0.09	0.63	−0.15	0.79*	0.97**	

Note: *Significant correlations = 0.01 < *p* < 0.05; **denotes *p* < 0.01

The predicted genetic gains of the hybrid clones at the six sites for different selections are presented in Table 7. The results are based on the assumption of the estimated repeatabilities and correlations from direct clonal selection and the related genetic responses in all the studied traits. The predicted genetic gains were identical in C for Pinggu and Zhengzhou (6.1%), while the Taiyuan site had the highest gain (8.4%). However, the gains in C were about twice the gains observed for FL. The selection for C led to equal gains in FW as direct selection for FW. The selection for DBH led to the gain in SV equal to that of the direct selection for SV but resulted in a slight decrease in C (Table 7). Among growth traits, selection for C resulted in the lowest gains in DBH and SV, however, it had a greater negative impact on SV.

**Table 7:** Expected response ($\varDelta G/\overline{X}\times 100$) in fiber properties and growth traits at the six sites for different selection criteria of the nine triploid hybrid clones

Selection criterion		Response (%)						
		FL	FW	FL/W	C	H	DBH	SV
Yanzhou	FL	**4.9**	2.2	3.3	6.6	–	–	–
	FW	3.6	**3.9**	−1.1	7.5	–	–	–
	FL/W	3.9	−0.9	**4.3**	1.2	–	–	–
	C	4.2	3.6	0.7	**7.5**	–	–	–
Gaotang	FL	**4.1**	2.7	–	5.2	3.7	6.6	13.7
	FW	3.8	**3.0**	–	7.3	1.8	−1.9	−3.9
	FL/W	–	–	–	–	–	–	–
	C	3.1	3.1	–	**7.8**	−0.1	−3.5	−7.7
	H	2.3	0.7	–	−0.1	**7.0**	6.7	17.6
	DBH	1.1	−0.6	–	−2.5	4.7	**9.8**	22.3
	SV	1.2	−0.5	–	−2.2	5.3	9.6	**22.7**
Xiangfen	FL	**3.2**	3.0	1.0	6.0	3.0	3.4	13.4
	FW	3.2	**3.3**	−0.5	7.2	−0.1	0.4	0.8
	FL/W	1.7	−0.7	**1.4**	−1.1	5.1	4.4	10.8
	C	2.7	3.1	−0.3	**7.4**	−1.9	−0.6	−5.2
	H	1.3	−0.1	1.6	−2.2	**5.8**	4.6	13.5
	DBH	1.3	0.2	1.4	−0.7	4.8	**4.7**	14.9
	SV	1.4	0.1	1.6	−1.7	5.3	4.5	**15.4**
Pinggu	FL	**3.8**	–	–	4.6	7.9	9.0	17.8
	FW	–	–	–	–	–	–	–
	FL/W	–	–	–	–	–	–	–
	C	2.9	–	–	**6.1**	2.9	−3.1	−4.7
	H	3.5	–	–	2.0	**6.0**	14.0	26.9
	DBH	2.1	–	–	−1.1	7.3	**15.5**	32.5
	SV	2.2	–	–	−0.9	7.6	17.4	**27.4**
Zhengzhou	FL	**3.3**	1.6	2.6	4.0	4.4	4.0	8.4
	FW	2.2	**2.6**	−1.0	5.7	−2.1	−4.6	−10.6
	FL/W	2.7	−0.8	**2.7**	−0.3	7.0	8.7	19.0
	C	2.3	2.4	−0.2	**6.1**	2.7	−1.8	−2.0
	H	1.8	−0.6	2.7	1.8	**9.6**	9.1	22.4
	DBH	1.4	−1.2	2.9	−1.0	7.8	**7.8**	24.6
	SV	1.4	−1.2	2.9	−0.6	9.0	11.5	**18.1**
Taiyuan	FL	**4.0**	2.6	2.3	6.6	3.7	2.7	9.3
	FW	3.5	**2.4**	0.7	7.4	1.7	−2.9	−2.7
	FL/W	3.3	0.8	**1.9**	2.8	4.3	7.9	18.8
	C	3.5	2.8	1.0	**8.4**	−0.6	−2.8	−4.5
	H	1.4	0.5	1.1	−0.4	**11.9**	8.1	24.0
	DBH	0.9	−0.7	1.9	−1.8	7.4	**11.3**	29.6
	SV	1.4	−0.3	1.9	−1.3	9.5	12.7	**28.2**

## Discussion

### Variations between sites

**Fig. 1:**
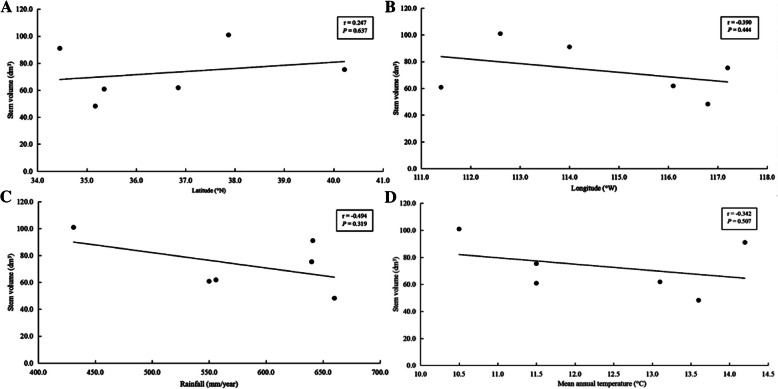
Correlations between stem volume and (**A**) latitude, (**B**) longitude, (**C**) rainfall, and (**D**) mean annual temperature

Site effects represent the response of trees to the combined influences of edaphic and regional climatic conditions [16]. Even though the current trials were not designed to separate these different effects, some conclusions can still be drawn [16–18]. The poor field performance in growth at Yanzhou may be owing to poor drainage (due to soil compression) and wet weather at the early stages of the experiment. Significant site effects were observed in the combined analysis of all traits (Table 2). Moreover, a positive correlation between SV and latitude, and negative correlations between SV and longitude, as well as rainfall and mean annual temperature, were observed (Fig. 1.), suggesting that edaphic and regional climatic conditions had significant effects on the growth traits. At all sites, the fiber properties of the triploid hybrid clones had less phenotypic plasticity than the growth traits, as evidenced by the lower CV values, indicating that the site impacts fiber properties less than the growth traits.

### Clonal variation and clone × site interaction

Former studies recorded that the effect of clone × site interactions on the growth traits at three clonal trials was greater than the clonal effect [4, 5]. Such a high interaction would lead to an increase in the complexity of testing [19, 20]. If the correlations between test and planting environments were alike and the objectives of an improvement program were restricted enough to allow selection for certain types of locations, then these interactions could be utilized. The impact of clonal effects on the growth traits in this study was, on the contrary, greater than the clone × site interactions when the number of testing sites was six. This suggests that an increase in the number of clonal testing sites helps improve the accuracy of clonal effects, thus reducing the complexity of clonal testing.

In this study, no significant positive type B genotypic correlations between genotypic values at the Xiangfen-Taiyuan sites for all studied traits were observed (Table 4), which suggests that a true G × E interaction exists. The differences between these sites may contribute to the clone × site interaction. However, the type B genotypic correlations for FL and C between the sites were mostly positive, which indicates that the fiber properties were more stable than the growth traits [21].

### Stability and reaction norms of clones

For forest tree breeding, it is highly important to predict the yield of a genotype in different environments. This concept of stability may be determined by the stability variances [22] and the regression coefficients [23]. In this study, the results indicated that each of the studied clones had various magnitudes of phenotypic plasticity and specific patterns of FL and SV at the six sites. The stability values for the FL of the triploid hybrid clones were different for the different methods. This could be partially explained by the different principles that are used to estimate the stability parameters [24–27]. High values of the Finlay-Wilkinson stability parameter, i.e., the regression coefficient of the clone values on location value, determine the clones that benefit from a productive location, while high intercept coefficients determine the clones that can grow well under limited resource conditions. As shown by the higher regression coefficient means of clone B304, B330, and B331 for SV, these clones have higher norms of reaction to the improvement in growth environments and higher phenotypic plasticity.

### Genotypic correlation between traits and clonal repeatability

Generally, an increase in tree growth was related to a slightly higher FL. Several previous studies have shown that FL increases with increasing tree growth [28–31]. In the present study, the genetic correlations between growth traits and FL were also positive at the six sites, indicating that triploid breeding of *Populus* not only improves the FL but also increases the tree growth.

Clonal repeatability of the fiber properties and growth traits across all six sites varied from medium to high (Table 2). For fiber properties, the estimated clonal repeatabilities ranged from 0.83 to 0.93, which was consistent with those that have been recorded in the literature [4, 5, 32]. This suggests that three clonal testing sites were enough to estimate quantitative parameters for fiber properties. However, clonal repeatabilities were estimated to be 0.76 for H, 0.85 for DBH, and 0.80 for SV, which were slightly higher than those estimated for the three clonal trials [5]. This implies that increasing the number of testing sites would help improve the accuracy of clonal repeatabilities of growth traits.

The analyses of the present study were based on nine genotypes and a relatively small number of testing sites measured over only five years. Therefore, the results from this study should be further verified by subsequent larger and longer trials. The implication of the G × E interaction for estimating the gain of genetically improved triploid hybrid clones of *P. tomentosa* should be further studied.

## Conclusions

The experiments and analyses lead to the conclusion that the clone and site have a highly significant effect (*P* < 0.001) for all studied traits. The clone × site interactions had a moderate effect on FW and FL/W, and a highly significant effect (*P* < 0.001) on FL, C, and tree growth. The stability parameter analysis showed that most of the triploid clones had higher reaction norms to the improvement in growth conditions and higher phenotypic plasticity. The estimated clonal repeatability of FW (0.93) was slightly higher than for C (0.91), FL (0.89), FL/W (0.83), H (0.85), DBH (0.76), and SV (0.80). Three clonal testing sites were good enough to estimate quantitative parameters of fiber properties. However, more than three clonal testing sites would help improve the accuracy of quantitative parameters of growth traits. The findings in this study were due to a certain number of triploid hybrid clones derived from several parents (*n* = 5), therefore, the implication of G × E interaction for calculating the gain of genetically improved triploid hybrid clones of *P. tomentosa* should be further investigated.

## Methods

### Experimental design

In the present study, the materials used were sampled from six triploid hybrid poplar clonal trials in northern China. These trials were established by Beijing Forestry University using cuttings of *P. tomentosa* on sandy loam with typical soil fertility. The clonal trials at Yanzhou, Gaotang, and Xiangfen were built in the spring of 2004 and the clonal trials at Pinggu, Taiyuan, and Zhengzhou were established in the spring of 2005. The characteristics of the six clonal experiments are shown in Table 8. The planted cuttings were 1.5–2.0 cm in diameter and 20–25 cm in length. The experiments were repeated three times in the nursery. The experiments included one diploid clone and nine triploid hybrid clones at each site (Table 9). The nine triploid hybrid clones were preselected among the triploids derived from five parent trees because they exhibited good growth rates [33]. The diploid clone (M1319) was an elite tree of *P. tomentosa* that grows faster. A randomized complete block design with three replicates was used in each clonal trial (240 trees per plot, resulting in a total of 720 trees per clone per trial). Ten clones were planted on rectangular plots at each location, and each plot contained 240 trees (4 × 60 trees) with 2 m × 3 m spacing. No thinning was conducted during the testing period.

**Table 8:** Location, climatic conditions, and description of six clonal trials

Site	Latitude (°N)	Longitude (°W)	Mean annual T (°C)	Rainfall (mm/year)	Number of clones	Sample trees
Yanzhou	35°10′	116°49′	13.6	660	10	54
Gaotang	36°51′	116°04′	13.1	556	10	54
Xiangfen	35°50′	111°21′	11.5	550	10	54
Pinggu	40°13′	117°12′	11.5	640	10	54
Zhengzhou	34°27′	113°57′	14.2	641	10	54
Taiyuan	37°52′	112°33′	10.5	431	10	54

**Table 9:** Identity and origin of the hybrid clones

No.	Clone identity	Parents	Level of ploidy	Sex
1	B301	(*P. tomentosa* × *P. bolleana*) × *P. tomentosa*	Triploid	♀
2	B302	(*P. tomentosa* × *P. bolleana*) × *P. tomentosa*	Triploid	♂
3	B303	(*P. tomentosa* × *P. bolleana*) × *P. tomentosa*	Triploid	♀
4	B304	(*P. tomentosa* × *P. bolleana*) × *P. tomentosa*	Triploid	♀
5	B305	(*P. tomentosa* × *P. bolleana*) × *P. tomentosa*	Triploid	♀
6	B306	(*P. tomentosa* × *P. bolleana*) × *P. tomentosa*	Triploid	♀
7	B312	(*P. tomentosa* × *P. bolleana*) × *P. tomentosa*	Triploid	♂
8	B330	(*P. alba* × *P. glandulosa*) × *P. tomentosa*	Triploid	♀
9	B331	(*P. alba* × *P. glandulosa*) × *P. tomentosa*	Triploid	♀
10	M1319	*P. tomentosa* × *P. tomentosa*	Diploid	♂

### Measurement of fiber properties and growth traits

A total of 360 trees were randomly selected from the six trial sites. Trees were collected randomly, 6 ramets (the 6 trees) of each clone were sampled fully at random per location. In the triploid poplar breeding program of Beijing Forestry University, the overall H and DBH of all tree samples were measured by workers at the age of 5 years. The SV of each sample was estimated according to the volume function of DBH and H used by Chen [34]. All sampled trees were shipped to Beijing Forestry University. For laboratory measurements, a 10-cm-thick stem disk was harvested from the breast height of each sample. Matchstick-sized wood samples (each sample implying two pairs of annual rings) were cut from the stem disks to evaluate the fiber properties, and then immersed in a boiling 1:1 (v/v) mixed solution of hydrogen peroxide and acetic acid. Afterward, FL, FW, and C were measured according to the methods described by Zhang et al. [4] using the Lorentzen & Wettre (LW) Fiber Tester (AB Lorentzen & Wettre, Kista, Sweden).

### Statistical analysis

When the main statistical analysis and the estimation of genetic statistics were conducted, the diploid clone (M1319) was not included. Thus, only the nine triploid clones were used in the main variance analysis and estimates of genetic parameters. Analyses of variance were performed according to the UNIVARIATE program of the SPSS software (SPSS for Windows, version 13, SPSS, Chicago, IL). Analysis of variance was used to analyze the variation between the ramets of the sample clones at a location based on the following linear model (Eq. (1)):

1 $$ {X}_{ik}=\mu +{C}_i+{\varepsilon}_{ik} $$

where *X*_*ik*_ represents the observation of the *k*th ramet of the *i*th clone; *μ* represents the general mean; C_*i*_ represents the effect attributed to the *i*th clone and *ε*_*ik*_ represents random error. The clone × replicate effects were not included in the linear model (Eq. (1)) as the six sampled trees per clone per location were randomly selected.

The clonal repeatabilities were calculated within a location as:

2 $$ {R}_c^2=\frac{{\hat{\sigma}}_c^2}{{\hat{\sigma}}_c^2+\frac{{\hat{\sigma}}_e^2}{k}} $$

and the clonal repeatability of the individual-tree (${R}_b^2$) was estimated according to the following equation:

3 $$ {R}_b^2={\hat{\sigma}}_c^2/\left({\hat{\sigma}}_c^2+{\hat{\sigma}}_e^2\right) $$

where *k* is the average value of tree samples per clone within a location; ${\hat{\sigma}}_c^2$ is the estimated variance of clone; ${\hat{\sigma}}_e^2$ is the variance among the ramets within the clones.

The six locations were jointly analyzed using the following linear model [17] (Eq. (4)):

4 $$ {X}_{ijk}=\mu +{C}_i+{L}_j+{C}_i{L}_j+{\varepsilon}_{ijk} $$

where *X*_*ijk*_ represents the performance of the *k*th ramet of the *i*th clone within the *j*th location; *μ* represents the general mean; *C*_*i*_ represents the effect attributed to the *i*th clone; *L*_*j*_ represents the effect attributed to the *j*th location; *C*_*i*_*L*_*j*_ represents the interaction between the *i*th clone and *j*th location, and *ε*_*ijk*_ represents random error. Only the location was regarded as a fixed effect and all other terms were regarded as random effects.

The clonal repeatabilities were estimated using the equation:

5 $$ {R}_c^2=\frac{{\hat{\sigma}}_c^2}{\frac{k_2{\hat{\sigma}}_c^2}{k_2}+\frac{k_1{\hat{\sigma}}_{Lxc}^2}{k_2}+\frac{{\hat{\sigma}}_e^2}{k_2}} $$

and the clonal repeatability of individual-trees was calculated across locations according to the following equation:

6 $$ {R}_b^2={\hat{\sigma}}_c^2/\left({\hat{\sigma}}_c^2+{\hat{\sigma}}_{Lxc}^2+{\hat{\sigma}}_e^2\right) $$

where *k*_1_ is the coefficient related to the variance caused by the clone × site interaction item (${\hat{\sigma}}_{Lxc}^2$); *k*_2_ is the coefficient related to the variance caused by the clonal variation (${\hat{\sigma}}_c^2$). The standard errors (SE) for the estimates of clonal repeatability were estimated according to the following equation [35]:

7 $$ SE\left({R}_c^2\right)=\sqrt{\frac{2{\left(1-{R}_c^2\right)}^2{\left[1+\left({k}_2-1\right){R}_c^2\right]}^2}{k_2\left({k}_2-1\right)N-1}} $$

where *N* is the number of tested clones. The SE for the clonal repeatability estimates of individual-tree were also estimated according to eq. (7), where ${R}_b^2$ was used instead of ${R}_c^2$.

The genetic correlation coefficients (*r*_*A(XY)*_) between traits at a location were computed using the equation [36]:

8 $$ {r}_{A\left(X,Y\right)}=\frac{{\hat{\sigma}}_{c\left(x,y\right)}}{\sqrt{{\hat{\sigma}}_{c(x)}^2{\hat{\sigma}}_{c(y)}^2}} $$

where ${\hat{\sigma}}_{c\left(x,y\right)}$ is the clonal component of covariance estimated among traits *x* and *y*; ${\hat{\sigma}}_{c(x)}^2$ is the clonal variance component estimated for trait x; ${\hat{\sigma}}_{c(y)}^2$ is the clonal variance component estimated for trait y. Similar to using the mean squares for estimating variance components, the cross-products were used to estimate the corresponding clone component of variance from the data collected from the same individual tree.

The approximate SE of genetic correlation estimates were estimated based on the following formula [9]:

9 $$ SE=\frac{1-{r}^2}{\sqrt{2}}\sqrt{\frac{{\hat{\sigma}}_{\left({R}_x^2\right)}{\hat{\sigma}}_{\left({R}_y^2\right)}}{R_x^2{R}_y^2}} $$

where *r* is the estimate of genetic correlation; ${R}_x^2$ is the estimate of the clonal repeatability of the trait *x*; ${R}_y^2$ is the estimate of the clonal repeatability of the trait *y*; ${\hat{\sigma}}_{\left({R}_x^2\right)}$ and ${\hat{\sigma}}_{\left({R}_y^2\right)}$ are the standard error for ${R}_x^2$ and ${R}_y^2$, respectively. Using Eq. (7) as the basis for the *t*-tests, the significance of the genetic correlations between the traits was tested.

The genetic correlation estimates among the same traits for pairs of locations were estimated to assess the contribution of each pair of treatments to the overall clone × environment. These type B genotypic correlations were computed according to the measurements of sample ramets at different locations from the same clones planted using the equation [37]:

10 $$ {r}_{B\left(X,Y\right)}=\frac{r_{P\left(x1,y2\right)}}{R_{C(x1)}{R}_{C(y2)}} $$

where *r*_*p(x1,y2)*_ is the coefficient of phenotypic correlation between the clonal means estimated between *x* measured at location 1 and *y* measured at location 2; *R*_*c(x1)*_ and *R*_*c(y2)*_ are the square roots of the mean repeatability of the clones x and y estimated at location 1 and 2, respectively.

The SPSS PROC CORR software was used to calculate the phenotypic correlations of the clonal means for each pair of sites, and the significance test was performed using the *F*-test.

In the present study, it is assumed that the selection intensity is equal to 1.271, which corresponds to selecting four clones from 18 or three clones from 14 [16]. According to the relevant response of the clonal selection in trait *x*, the expected genetic gain in trait *y ΔG*_*y*_ can be predicted using the formula [9]:

11 $$ \varDelta {G}_y={i}_x\sqrt{R_x^2}{\sigma}_y{r}_{xy} $$

where *i*_*x*_ (*i*_*x*_ = 1.271) is the selection intensity; ${R}_x^2$ is the clonal repeatabilities for trait *x*; *σ*_*y*_ is the standard deviation of clonal for trait *y*; *r*_*xy*_ is the genetic correlation coefficients between trait *x* and *y* (*r*_*xy*_ ≤ 1).

### Estimation of stability parameters

To evaluate the stability of the individual clones across the six experimental locations, the stability variances were estimated using the Shukla model [22], where lower values indicate higher stability, and the significance test was conducted using the *F*-test. Subsequently, the stability of the individual clones across locations was also estimated using the Finlay and Wilkinson stability parameter [23]. The triploid clones were grouped according to the size of their regression coefficients (*b*_*i*_), less than, equal to, or > 1. Clones with *b*_*i*_ > 1 are more adapted to favorable growing conditions, those with *b*_*i*_ < 1 are adapted to unfavorable environmental conditions, and those with *b*_*i*_ equal to 1 have an average adaptation to all environments [23].

## Data Availability

All data generated or analysed during this study are included in this published article.

## Abbreviations

CV *p* %: coefficients of phenotypic variation
FL: fiber length
FW: fiber width
FL/W: fiber length/width
C: coarseness
H: tree height
DBH: diameter at breast height
SV: stem volume
${R}_b^2$: repeatability of individual-tree clonal
${R}_c^2$: repeatability of clonal means
SE: standard errors
YZ: Yanzhou
GT: Gaotang
XF: Xiangfen
PG: Pinggu
ZZ: Zhengzhou
TY: Taiyuan
*F*: Fisher’s criteria
*P*: probability levels
*t*: Student’s criteria
*b*_*i*_: regression coefficient
